# Avoidant symptoms in PTSD predict fear circuit activation during multimodal fear extinction

**DOI:** 10.3389/fnhum.2013.00672

**Published:** 2013-10-17

**Authors:** Rebecca K. Sripada, Sarah N. Garfinkel, Israel Liberzon

**Affiliations:** ^1^Department of Psychiatry, University of MichiganAnn Arbor, MI, USA; ^2^Veterans Affairs Center for Clinical Management Research, Department of Veterans Affairs Healthcare SystemAnn Arbor, MI, USA; ^3^Department of Psychiatry, Brighton and Sussex Medical SchoolBrighton, UK

**Keywords:** fear conditioning, avoidance, posttraumatic stress disorder, fMRI, neuroimaging, amygdala, hippocampus

## Abstract

Convergent evidence suggests that individuals with posttraumatic stress disorder (PTSD) exhibit exaggerated avoidance behaviors as well as abnormalities in Pavlonian fear conditioning. However, the link between the two features of this disorder is not well understood. In order to probe the brain basis of aberrant extinction learning in PTSD, we administered a multimodal classical fear conditioning/extinction paradigm that incorporated affectively relevant information from two sensory channels (visual and tactile) while participants underwent fMRI scanning. The sample consisted of fifteen OEF/OIF veterans with PTSD. In response to conditioned cues and contextual information, greater avoidance symptomatology was associated with greater activation in amygdala, hippocampus, vmPFC, dmPFC, and insula, during both fear acquisition and fear extinction. Heightened responses to previously conditioned stimuli in individuals with more severe PTSD could indicate a deficiency in safety learning, consistent with PTSD symptomatology. The close link between avoidance symptoms and fear circuit activation suggests that this symptom cluster may be a key component of fear extinction deficits in PTSD and/or may be particularly amenable to change through extinction-based therapies.

## INTRODUCTION

Posttraumatic stress disorder (PTSD) is a debilitating anxiety disorder that afflicts approximately 7 percent of the general population (Kessler et al., 2005). PTSD is characterized by three symptom clusters: reexperiencing, hyperarousal, and avoidance symptoms (APA, 2000). The avoidance cluster includes avoidance of internal and external reminders of the trauma, failure to recall important aspects of the trauma, loss of interest in significant activities, subjective detachment or estrangement from others, restricted range of affect, and sense of foreshortened future (APA, 2000). Some studies suggest that avoidance symptoms track the diagnosis of PTSD better than either of the other two symptom clusters (North et al., 1999). In addition, clinical research indicates that for individuals with PTSD, avoidance symptoms may be the most detrimental symptoms to psychosocial functioning (Hendrix et al., 1998; Riggs et al., 1998; Ruscio et al., 2002; Kuhn et al., 2003; Samper et al., 2004; Lauterbach et al., 2007; Solomon and Mikulincer, 2007; Malta et al., 2009) and quality of life (Lunney and Schnurr, 2007; Schnurr and Lunney, 2008). Furthermore, early avoidance symptoms may predict subsequent PTSD development (Bryant et al., 2000; North et al., 2012). These multiple lines of evidence suggest that avoidant symptoms might signify a key process in PTSD pathophysiology.

In parallel, convergent evidence suggests that PTSD is associated with various abnormalities in fear associated learning, including greater acquisition of conditioned fear, overgeneralization of conditioning, impaired inhibitory learning, and impaired extinction (Orr et al., 2000; Lissek et al., 2005; Milad et al., 2008, 2009; Jovanovic et al., 2009, 2010; Rougemont-Bucking et al., 2011; Mahan and Ressler, 2012; Lommen et al., 2013). It has been suggested that deficits in fear associated learning may play a role in the development (see Lommen et al., 2013) and maintenance (Mahan and Ressler, 2012) of PTSD, and that abnormalities in the extinction and/or retention of conditioned fear may be particularly salient for the persistence of fear memories in PTSD (Milad et al., 2008, 2009). Few studies to date have probed the neural circuitry underlying fear extinction deficits in PTSD, but the existing evidence suggests key roles for amygdala, hippocampus, and vmPFC in this process (Milad et al., 2005, 2007, 2009; Mahan and Ressler, 2012).

Conceptually, deficits in fear-associated learning have been hypothesized to contribute to the development and maintenance of reexperiencing and hyperarousal symptoms. However, the link between fear-associated learning deficits and other key components of PTSD pathophysiology, such as avoidance symptoms, is not well understood. Animal models suggest that avoidance may stem from fear extinction deficits. For instance, Chen et al. (2012) demonstrated that rats displaying greater fear after conditioning go on to exhibit greater behavioral avoidance over a 4 week period. It is also possible that avoidance symptoms may exacerbate fear extinction deficits by reducing the frequency with which individuals come in contact with feared stimuli, thus providing less opportunity for extinction to occur. For instance, socially anxious individuals with more severe avoidance in early treatment experience greater subsequent fear in later treatment (Aderka et al., 2013). However, no research has investigated the neurobiological underpinnings of this phenomenon.

In order to probe the brain basis of the link between avoidance and aberrant extinction learning in PTSD, we administered a multimodal classical fear conditioning/extinction paradigm that incorporated affectively relevant information from two sensory channels (visual and tactile) in an fMRI environment. Mild shock was used as the unconditioned stimulus (US), and colored lights were used as the conditioned stimuli (CS+ and CS-). We have previously demonstrated that PTSD patients exhibit impaired extinction recall and greater return of extinguished fear when trauma-relevant stimuli are presented, and that these abnormalities are particularly associated with avoidance symptoms (Garfinkel et al., unpublished). Thus, the current study sought to investigate whether individual differences in avoidance symptoms might influence extinction learning. We hypothesized that in response to conditioned stimuli (CS’s) and context, individuals with more severe avoidance symptoms would demonstrate greater activity in brain networks related to emotion processing and fear expression.

## MATERIAL AND METHODS

### SUBJECTS

Fifteen right-handed OEF/OIF veterans with PTSD were recruited from the Ann Arbor Veterans Affairs PTSD Clinic. Mean age was 27.3 (SD = 4.5). Ten patients were married and five were single. Twelve patients were Caucasian, one was Asian, one was African-American, and one was Hispanic. Participants were included as part of a larger sample (Sripada et al., 2012a, 2012b; Garfinkel et al., unpublished) that also included healthy combat-exposed controls. All participants received comprehensive psychiatric assessment with the Mini-International Neuropsychiatric Interview (Sheehan et al., 1998) and the Clinician-Administered PTSD Scale (CAPS; Blake et al., 1995). Mean CAPS score was 75.9 (SD = 17.2). All combat exposure (including index trauma for PTSD participants) took place within 5 years prior to study enrollment. Clinical interviews were performed by experienced masters- or doctoral-level clinicians with extensive training in the CAPS, at a subspecialty clinic specializing in PTSD. Exclusion criteria were as follows: (a) psychosis, (b) history of traumatic brain injury, (c) alcohol or substance abuse or dependence in the past 3 months, (d) any psychoactive medication other than sleep aids, (e) left-handedness, (f) presence of ferrous-containing metals within the body, and (g) claustrophobia. Seven participants also met diagnostic criteria for depression, and one had comorbid panic disorder; however, PTSD was always the primary diagnosis. Two participants were using low-dose trazodone as a sleep aid; no other psychiatric medications were permitted. After a complete description of the study was provided to the participants, written informed consent was obtained. The study was approved by the institutional review boards of the University of Michigan Medical School and the Ann Arbor VA Healthcare System. All procedures took place between August 2008 and July 2010.

### TASK

Participants were fear conditioned in a modified version of Milad et al.’s (2005, 2007) paradigm. The CS’s were colored lights (pink and blue), presented on a background of an office or library setting (context). The US was an electric shock (500 ms duration pulse sequence) delivered to the index and middle fingers, titrated individually to the level defined as “highly annoying but not painful” (Orr et al., 2000).

**FIGURE 1:**
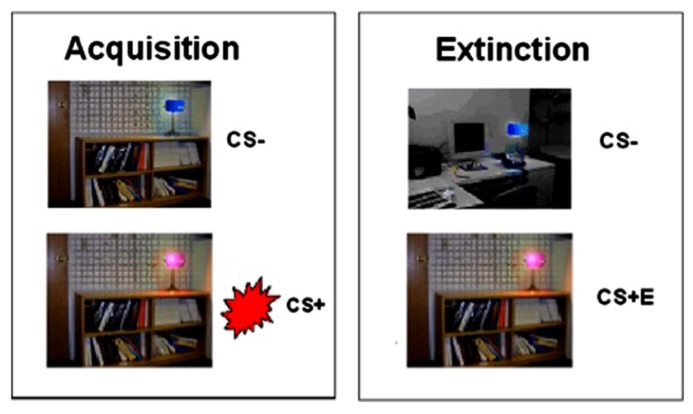
**Fear conditioning and extinction procedures**. Fear conditioning contingencies were established during acquisition, followed by extinction of the CS+ (to form CS+E).

Habituation, fear acquisition, and fear extinction all occurred within the scanner in three separate functional runs. Prior to each run, participants were informed that they could receive a shock at any time (see Milad et al., 2007). Habituation involved 12 presentations of the context plus light pairings, and ensured that participants became familiar with stimuli and contexts. During fear acquisition, one context (either the office or library) was presented, counterbalanced on a between-subjects basis. This context remained on the screen for 2–7 s, followed by activation of the light (either pink or blue) for a further 2–7 s, ensuring that the epoch for each context and context + light paring amounted to 9 s in total. For the CS+, the US was delivered at 60% contingency (10 out of 16 trials), to coincide with CS offset. The other CS was presented 16 times, and was never associated with shock (forming the CS-). The 16 CS- trials were interleaved with the 16 CS+ trials. Each trial was followed by the presentation of a white fixation cross on a black background, jittered for a duration of 12–18 s. Fear extinction followed fear acquisition, and involved a switch in context (from office to library or vice versa). During extinction, the stimuli formerly associated with shock (CS+) were presented in the absence of shock (CS+E), interspersed with presentations of the CS- (see **Figure 1**). 16 presentations of the CS+E were interleaved with 16 CS- presentations. The 32 trials of fear acquisition and 32 trials of extinction learning were blocked into the first 16 (early) trials and the last 16 (late) trials. In order to isolate successfully acquired (fully learned) conditioning, we restricted our analysis of the acquisition phase to the *late* acquisition phase. Conversely, to maintain a focus on extinction learning rather than extinction retention, our analysis of the extinction phase was restricted to *early* extinction.

### fMRI DATA ACQUISITION

Scans were collected on a 3.0 Tesla General Electric Signa® Excite^TM^ scanner (Milwaukee, WI, USA). After subjects were positioned in the scanner, a T1-weighted low resolution structural image was acquired approximately parallel to the AC-PC line [gradient recall echo sequence (GRE), repetition time (TR) = 250 ms, echo time (TE) = 5.7 ms, flip angle (FA) = 90^°^, 2 averages, field of view (FOV) = 22 cm, matrix = 256 × 256, slice thickness = 3 mm, 40 axial slices to cover the whole brain], which was identical to the prescription of the functional acquisitions. Functional images were acquired with a T2*-weighted, reverse spiral acquisition sequence (gradient recall echo, TR = 2000 ms, TE = 30 ms, FA = 90^°^, FOV = 22 cm, matrix = 64 × 64, slice thickness = 3 mm with no gap, 40 axial slices to cover the whole brain, acquisition voxel size = 3 × 3 × 3 mm) which has been shown to minimize signal drop-out in regions such as ventral striatum and orbitofrontal cortex that are vulnerable to susceptibility artifact (Glover and Law, 2001). The intermediate template and fMRI images were acquired using a GE Quadrature sending and receiving head coil. Four initial volumes were discarded from each run to allow for equilibration of the scanner signal. A high-quality T1-weighted structural image was obtained with a 3-D volume inversion recovery fast spoiled gradient recalled echo (IR-FSPGR) protocol (TR = 12.3 ms, TE = 5.2 ms, FA = 9^°^, TI = 650 ms, FOV = 26 cm, matrix = 256 × 256 for in-plane resolution of 1 mm; slice thickness = 1 mm with no gap, 160 contiguous axial slices to cover the whole brain), using an eight-channel GE phase array receiving head coil. E-prime was used to present stimuli (Psychology Software Tools, Pittsburgh, PA, USA). Participants wore glasses with built-in mirrors (NordicNeuro Labs) in order to view the projected stimuli inside the scanner.

### PREPROCESSING OF fMRI DATA

An initial series of preprocessing steps was carried out. First, we removed k-space outliers in raw data that were two standard deviations away from the mean and substituted them with the average value from neighboring voxels. Next, a B_0_ field map was used in the reconstruction of the images to remove the distortions that resulted from magnetic field inhomogeneity [IEEE-TIME, 10:629-637, 1991]. The variance due to physiological responses (i.e., cardiac and respiratory sources) was removed using regression (Glover et al., 2000). Additional preprocessing and image analysis was performed in SPM5 (Wellcome Department of Cognitive Neurology, London, UK; ). The T2 overlay was co-registered to the functional images, and then the high-resolution T1 image was co-registered to overlay. T1 images were normalized to the scalped T1 template and the functional volumes were normalized to the Montreal Neurological Institute (MNI) template using the previously computed transformation matrix. Images were smoothed using an isotropic 8 mm full-width-half maximum (FWHM) Gaussian kernel.

### ANALYSIS

fMRI comparisons of interest were implemented as linear contrasts. Realignment parameters were added as covariates of no interest at the first level. Z-score images from individual analyses were entered into second-level random-effects analyses (one-sample and two-sample *t*-tests) implemented in SPM5. Second-level maps were thresholded at *p* < 0.001, cluster-level corrected for multiple comparisons via family wise error correction. Regions of interests (ROIs) were selected from a systematic review of fMRI fear conditioning studies (Sehlmeyer et al., 2009), and defined using the automated anatomical labeling atlas (AAL). They included amygdala, hippocampus, vmPFC (bilateral medial orbital frontal gyrus), dmPFC (bilateral superior medial frontal gyrus), and insula. Only the clusters within the regions of interest that survived family wise error small volume correction were extracted and used for further analysis. Symptom severity was assessed via the CAPS, which consists of three subscales (reexperiencing symptoms, avoidance symptoms, and hyperarousal symptoms) that are summed for a total score. Bivariate correlations were computed between CAPS score and the extracted BOLD signal during fear acquisition and fear extinction.

## RESULTS

Subjects were successfully fear conditioned, as reported elsewhere (Garfinkel et al., unpublished). As predicted, fear conditioning activated a network of fear processing regions in response to CS+ presentation in both PTSD patients and Combat-exposed Controls. There were no between-group differences in brain activation patterns or skin conductance responses during the conditioning phase (Garfinkel et al., unpublished).

### CORRELATIONS WITH AVOIDANCE SYMPTOMS

#### FEAR ACQUISITION

To explore whether avoidance symptoms were associated with differential neural activation patterns during conditioning, the CAPS avoidance subscale was entered as a regressor in a whole brain analysis during the fear acquisition phase. During CS+ (as compared to CS-) greater CAPS avoidance was associated with greater activity in right hippocampus ([33, -27, -9], k = 9, z = 3.62, p = 0.025, SVC; see **Table 1**). This correlation remained significant after controlling for levels of reexperiencing and hyperarousal symptoms. No other significant associations were observed.

**Table 1:** Correlations with CAPS avoidance symptoms.

Contrast map and brain region	Cluster size	MNI coordinates (*x*, *y*, *z*)	Analysis (*z*)
Fear acquisition			
CS+ > CS-			
** Right hippocampus**	**9**	**33, -27, -9**	**3.62**
Fear extinction			
Context presentation > fixation			
Right superior temporal gyrus/insula	80	45, -12, -27	3.77
Left superior temporal gyrus	67	-51, 6, -9	3.64
**Left insula**	**21**	**-48, 6, -9**	**3.52**
Right middle temporal gyrus	61	42, -60, -6	3.94
Left middle temporal gyrus	61	-63, -63, 6	3.72
Left insula/thalamus	76	-27, -30, 12	3.83
**Left hippocampus**	**3**	**-21, -30, -3**	**3.42**
Right caudate	79	15, 21, 3	3.73
**Right amygdala**	**1**	**24, 3, -24**	**3.22**
Fear extinction			
CS+ > CS-			
Left inferior/middle temporal gyrus	185	-51, -6, -36	4.07
**Left insula**	**2**	**-39, -18, -3**	**3.45**
Left cerebellum	232	-9, -87, -33	4.17
Left inferior temporal gyrus	154	-72, -30, -12	4.67
Right middle temporal gyrus	333	60, -54, -6	5.11
Left superior temporal gyrus	162	-63, -51, 21	4.15
Right precuneus	184	12, -75, 48	3.72
**Right amygdala**	**5**	**24, 3, -24**	**3.89**
**Medial orbital frontal gyrus**	**14**	**3, 45, -6**	**3.35**
**Superior medial frontal gyrus**	**36**	**-6, 30, 57**	**3.77**
**Left hippocampus**	**17**	**-24, -21, -12**	**3.79**
Fear Extinction			
Fixation after CS+ > fixation after CS-			
Right fusiform/parahippocampal gyrus	318	36, -12, -30	5.03
**Right amygdala**	**9**	**27, 3, -27**	**3.62**
Left inferior orbital frontal gyrus	532	-45, 21, -12	4.47
**Left insula (anterior)**	**70**	**-39, 15, -12**	**4.35**
**Medial orbital frontal gyrus**	**11**	**-6, 21, -15**	**3.40**
Left insula/middle temporal gyrus	230	-69, -27, -12	3.74
**Left insula (posterior)**	**52**	**-36, -18, 6**	**3.63**
Right superior temporal pole	77	54, 18, -15	3.68
**Right insula**	**11**	**42, 6, -6**	**3.66**
Right hippocampus/parahippocampal gyrus	134	36, -30, -12	3.90
**Right hippocampus**	**26**	**36, -30, -12**	**3.90**
Supplementary motor area	255	-3, 9, 72	3.74
Right precentral gyrus	102	27, -24, 75	3.53

*Regions of interest (ROIs) in **bold**; significant at *p* < 0.05, family wise error corrected for multiple comparisons across the ROI. All other activations are presented at *p* < 0.001, cluster-level corrected for multiple comparisons via family wise error correction. MNI, Montreal Neurologic Institute.

#### FEAR EXTINCTION

**FIGURE 2:**
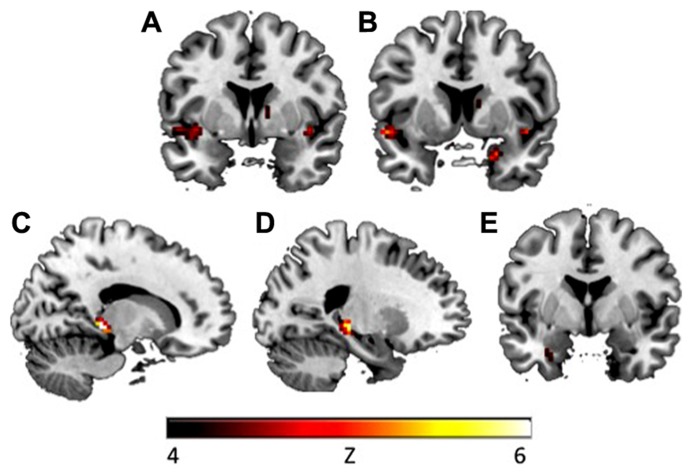
**During context presentation in the early extinction phase, CAPS avoidance symptoms were associated with increased activation in (A)** left insula (*y* **=** 2) and **(B)** right amygdala (*y* **=** 7). During context presentation, CAPS total symptoms were associated with increased activation in **(C)** right hippocampus (*x* = 15), **(D)** left hippocampus (*x* = -22), and **(E)** left amygdala (*y* = -1). Activations presented at *p* < 0.00005. CAPS, clinician-administered PTSD scale.

**FIGURE 3:**
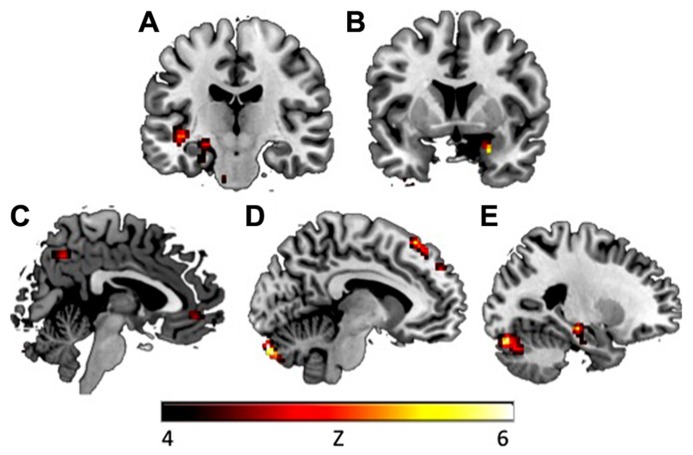
**During CS+** **presentation in the early extinction phase, CAPS avoidance symptoms were associated with increased activation in (A) left insula (*y* = -18), (B) right amygdala (*y* = 5), (C) vmPFC (*x* = 3), (D) dmPFC (*x* = -8), and (E) left hippocampus (*x* = -24).** Activations presented at *p* < 0.00005. vmPFC, ventromedial prefrontal cortex; dmPFC, dorsomedial prefrontal cortex.

**FIGURE 4:**
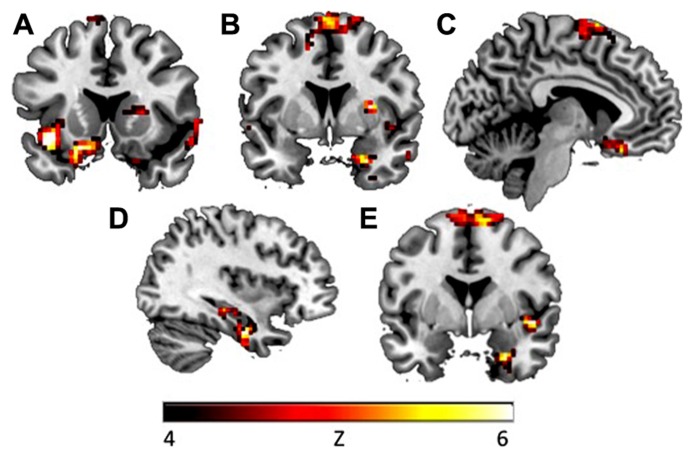
**During fixation after CS+ in the early extinction phase, CAPS avoidance symptoms were associated with increased activation in (A) left insula (*y* = -15), (B) right amygdala (*y* = 3), and (C) vmPFC (*x* = -6).** During fixation after CS+, CAPS total symptoms were associated with increased activation in (D) right hippocampus (*x* = 36), (E) right insula and right amygdala (*y* = 0). Activations presented at *p* < 0.00005.

To explore whether avoidance symptoms were associated with differential activation patterns during extinction learning, the CAPS avoidance subscale was entered as a regressor in a whole brain analysis during the fear extinction phase. During context presentation prior to CS presentation (as compared to fixation), avoidance was associated with greater activity in left hippocampus ([-21, -30, -3], *k* = 3, *z* = 3.42, *p* = 0.047, SVC), left insula ([-48, 6, -9], *k* = 21, *z* = 3.52, *p* = 0.05, SVC), and right amygdala ([24, 3, -24], *k* = 1, *z* = 3.22, *p* = 0.025, SVC; see **Figure 2**). Correlations with insula and amygdala remained significant after controlling for other PTSD symptom clusters. During CS+E (as compared to CS-), greater avoidance was associated with greater activity in right amygdala ([24, 3, -24], *k* = 5, *z* = 3.89, *p* = 0.002, SVC), vmPFC ([3, 45, -6], *k* = 14, *z* = 3.35, *p* = 0.05, SVC), dmPFC ([-6, 30, 57], *k* = 36, *z* = 3.77, *p* = 0.049, SVC), left insula ([-39, -18, -3], *k* = 2, *z* = 3.45, *p* = 0.05, SVC), and left hippocampus ([-24, -21, -12], *k* = 17, *z* = 3.79, *p* = 0.011, SVC; see **Figure 3**). The correlation with left hippocampus remained significant after controlling for other PTSD symptom clusters. Immediately following CS+E, during the period that involved shock administration while in the acquisition phase, greater avoidance was associated with greater activity in right amygdala ([27, 3, -27], *k* = 9, *z* = 3.62, *p* = 0.007, SVC), right insula ([42, 6, -6], *k* = 11, *z* = 3.66, *p* = 0.038, SVC), right hippocampus ([36, -30, -12], *k* = 26, *z* = 3.9, *p* = 0.009, SVC), left anterior insula ([-39, 15, -12], *k* = 70, *z* = 4.35, *p* = 0.004, SVC), left posterior insula ([-36, -18, 6], *k* = 52, *z* = 3.63, *p* = 0.042, SVC), and a trend for vmPFC ([33, -27, -9], *k* = 11, *z* = 3.4, *p* = 0.06, SVC; see **Figure 4**). Correlations with left and right insula remained significant after controlling for other PTSD symptom clusters. Whole brain activations are reported in **Table 1**.

### CORRELATIONS WITH CAPS TOTAL

To explore whether total PTSD symptoms were associated with differential activation patterns during fear acquisition and extinction learning, correlations were also computed between BOLD signal and CAPS total score. During extinction, in response to context presentation prior to CS, CAPS total was associated with greater activity in right hippocampus (2 clusters: [15, -36, 0], k = 4, z = 3.71, p = 0.017, SVC; [18, -33, -2], k = 2, z = 3.42, p = 0.039, SVC), left hippocampus (2 clusters: [-24, -30, -3], k = 23, z = 3.93, p = 0.011, SVC; [-27, -15, -12], k = 2, z = 3.45, p = 0.042, SVC), and left amygdala ([-30, 0, -27], k = 1, z = 3.21, p = 0.021, SVC; see **Table 2**; **Figure 2**). Left (p = 0.015, SVC) and right (p = 0.032, SVC) hippocampal activity was also significantly correlated with the sum of reexperiencing and hyperarousal symptom clusters (i.e., total CAPS score minus avoidance subscale). Immediately following CS+E, CAPS total was associated with greater activity in right amygdala (2 clusters: [33, -3, -27], k = 4, z = 3.35, p = 0.015, SVC; [27, 3, -27], k = 1, z = 3.10, p = 0.029, SVC), right hippocampus (2 clusters: [36, -9, -27], k = 37, z = 3.83, p = 0.012, SVC; [21, -36, 6], k = 6, z = 3.31, p = 0.05, SVC), and right insula (2 clusters: [45, 0, -6], k = 32, z = 4.39, p = 0.003, SVC; [33, -15, 6], k = 4, z = 3.59, p = 0.044, SVC; see **Figure 4**). Whole brain activations are reported in **Table 2**.

**Table 2:** Correlations with CAPS total symptoms.

**Contrast map and brain region**	**Cluster size**	**MNI coordinates (*x, y, z*)**	**Analysis (*z*)**
Fear extinction			
Context presentation > fixation			
**Right hippocampus (posterior)**	**4**	**15, -**36, 0	**3.71**
**Right hippocampus (anterior)**	**2**	**18, -**33, -2	**3.42**
**Left hippocampus (posterior)**	**23**	**-**24, -30, -3	**3.93**
**Left hippocampus (anterior)**	**2**	**-**27, -15, -12	**3.45**
**Left amygdala**	**1**	**-**30, 0, -27	**3.21**
Fear extinction			
Fixation after CS+ > fixation after CS-	****	****	****
Right fusiform/parahippocampal gyrus	168	36, **-**9, **-**30	4.4
**Right hippocampus (anterior)**	**37**	**36, -**9, -27	**3.83**
**Right amygdala (lateral)**	**4**	**33, -**3, -27	**3.35**
**Right amygdala (medial)**	**1**	**27, 3, -**27	**3.10**
Left cerebellum/fusiform gyrus	97	**-**24, **-**42, **-**15	3.47
Supplementary motor area	256	9, **-**3, 69	3.90
**Right hippocampus (posterior)**	**6**	**21, -**36, 6	**3.31**
**Right insula (anterior)**	**32**	**45, 0, -**6	**4.39**
**Right insula (posterior)**	**4**	**33, -**15, 6	**3.59**

*Regions of interest (ROIs) in **bold**; significant at *p* < 0.05, family wise error corrected for multiple comparisons across the ROI. All other activations are presented at *p* < 0.001, cluster-level corrected for multiple comparisons via family wise error correction. MNI, Montreal Neurologic Institute.

### CORRELATIONS WITH OTHER SYMPTOM CLUSTERS

For completeness, we also conducted an exploratory analysis to investigate whether reexperiencing or hyperarousal symptoms were associated with unique activation patterns during fear acquisition and extinction learning. During extinction, in response to context presentation prior to CS, CAPS hyperarousal was associated with greater activity in left hippocampus ([-24, -24, -12], *k* = 27, *z* = 4.17, *p* = 0.004, SVC). Reexperiencing symptoms were not associated with differential activation in any ROI.

### TIME COURSE OF ROI ACTIVATION

We implemented a series of repeated-measures ANOVAs to investigate the degree to which the different stages of extinction (context presentation, CS+E presentation, and immediate aftermath of CS+E presentation) activated key limbic and prefrontal regions. This analysis revealed that dmPFC activation varied by stage (*F*(26,2) = 3.82, *p* = 0.035). *Post-hoc* LSD tests showed that dmPFC exhibited greater activation during context processing (*M* = 0.26, *SD* = 0.67) than during CS+E presentation (*M* = -0.47, *SD* = 1.12; *p* = 0.048) or during the immediate aftermath of CS+E presentation (*M* = -0.16, *SD* = 0.72; *p* = 0.045). No other ROI demonstrated significantly different activation across stages.

## DISCUSSION

In this fMRI study, we investigated the neural underpinnings of the link between fear extinction learning and avoidant symptoms in PTSD. We found that amongst individuals with PTSD, greater avoidance symptomatology was associated with greater activation in emotion processing circuits in response to conditioned cues and contextual information, both during fear acquisition and fear extinction. This pattern was observed during presentation of context immediately prior to the CS (i.e., “context alone”), during the presentation of the CS+E, and immediately following the CS+E. Correlations with insula, amygdala, and hippocampus survived after controlling for other PTSD symptom clusters. Heightened responses to previously conditioned stimuli in individuals with more avoidant symptoms or more severe PTSD could indicate a deficiency in safety learning, consistent with PTSD symptomatology. The close link between avoidance symptoms and fear circuit activation suggests that this symptom cluster may be a key component of fear extinction deficits in PTSD and/or may be particularly amenable to change through extinction-based therapies.

The multimodal nature of this experiment enhanced its applicability to PTSD. Research demonstrates that multimodal trauma experiences may exacerbate PTSD symptoms, but also that multimodal treatment can enhance efficacy. For instance, extending the duration of context presentation (including tactile, visual, and olfactory cues) before foot-shock administration (in mice) increases generalization and avoidance (Sauerhofer et al., 2012). This finding was interpreted to suggest that fostering multimodal learning enhances conditioning (Sauerhofer et al., 2012). Conversely, emerging evidence suggests that individuals with high avoidance may benefit more from treatment when it incorporates multisensory trauma cues, which provides less opportunity for further avoidance (Rizzo et al., 2009; Norrholm and Jovanovic, 2010). For instance, in virtual reality exposure therapy, patients are immersed in simulations of trauma-relevant environments that allow for precise control of stimulus conditions. Directly delivering these multimodal cues can help circumvent clinical avoidance. Changes in PTSD symptom clusters, and particularly symptoms in the avoidant cluster, may be the key mechanism of PTSD treatment efficacy (Monson et al., 2012). Our paradigm is inherently multimodal because it involves manipulation of context (visual) and conditioned cues (tactile). Thus, it may be a more effective PTSD probe than unimodal paradigms. That avoidance symptoms were extensively correlated with brain activation while the other symptom clusters were not suggests that the multimodal nature of the paradigm was particularly effective at drawing out avoidant tendencies.

The current study suggests that avoidance symptoms are associated with hyperactivity in a variety of regions key to emotion processing and extinction learning (Sehlmeyer et al., 2009), including hippocampus, amygdala, insula, and medial prefrontal regions. In our data, avoidance symptoms were associated with greater hippocampal activity across both fear acquisition and fear extinction phases. Correlations between avoidance and hippocampal activity were observed during the presentation of context alone, during the presentation of previously conditioned cues, and immediately following the presentation of previously conditioned cues (during the period in which participants were shocked while in the acquisition phase). These findings are consistent with the role of hippocampus in contextual information processing (Maren et al., 2013) and with its role in “binding” contextual information with fear cues (Fanselow, 2000; Maren, 2001). Hippocampal activation was also associated with greater overall symptom severity, even after controlling for avoidance symptoms. This finding could help explain previous reports that PTSD patients exhibit greater hippocampal activity than healthy controls during fear acquisition and extinction learning (Bremner et al., 2005) or during non-fear related encoding (Werner et al., 2009). Interestingly, Milad et al. (2009) report *reduced* hippocampal activity in PTSD patients during extinction recall, which typically occurs the day after the conditioning and extinction phases. Conversely, in the present study, we found associations with avoidance during extinction *learning*, during the first 16 trials of extinction. This may reflect enhanced encoding or processing of conditioned associations formed during the acquisition phase in individuals with higher avoidance. This would suggest that higher avoidance is not only related to the expression of acquired fear, but also to fear learning. It is also possible that higher hippocampal activation in high avoidance patients reflects emotional rather than memory processing. Indeed, it has been demonstrated that anterior hippocampal regions in humans (which are analogous to ventral hippocampal regions in rodents; Moser and Moser, 1998), are involved in affect processing (Fanselow and Dong, 2010).

Avoidance symptom severity was also positively associated with amygdala and insula activity. These associations were present during context alone, during the presentation of previously conditioned cues, and immediately following conditioned cues. This too is consistent with previous animal and human findings. During extinction, high-anxious rats show hyperactivation (increased c-Fos expression) of the central nucleus of the amygdala (Muigg et al., 2008). Similarly, neuroimaging studies of individuals with PTSD report amygdala hyperactivity (Milad et al., 2009) and insula hyperactivity (Bremner et al., 2005) during extinction learning. Greater amygdala and insula activation during extinction is also correlated with trait anxiety (Barrett and Armony, 2009; Sehlmeyer et al., 2011). Insula and amygdala are key regions in salience detection and anticipation of negative events (Armony and LeDoux, 1997; Paulus and Stein, 2006). These regions are also associated with negative emotion production in PTSD, more generally (Shin and Liberzon, 2010). Greater activity in these emotion generation regions could thus reflect hyperactive fear responding to signals previously paired with negative outcomes. It could also reflect a failure to encode safety signals, or failure to adapt to or integrate new contextual information into previously learned contingencies (Liberzon and Sripada, 2008; Garfinkel and Liberzon, 2009).

We also found that avoidance symptom severity correlated with greater dmPFC and vmPFC activation. Greater dmPFC activity is consistent with previous findings of dmPFC/dACC hyperactivity in PTSD. For instance, Milad et al. (2009) report greater dACC activity during extinction recall in patients with PTSD, and Rougemont-Bucking et al. (2011) report exaggerated dACC activation in response to context presentation during late conditioning and early extinction. Other studies support that dmPFC/dACC hyperactivity in PTSD is also present during cognitive interference tasks such as oddball tasks (Bryant et al., 2005; Felmingham et al., 2009), Stroop tasks (Shin et al., 2007) and the Multi-Source Interference Task (Shin et al., 2011). The greater vmPFC activity found in highly avoidant patients, on the other hand, seemingly diverges from some previous reports of hypoactive vmPFC in PTSD (Bremner et al., 2005; Milad et al., 2009; Rougemont-Bucking et al., 2011). However, Barrett and Armony (2009) report that greater trait anxiety is associated with greater vmPFC activity during extinction. In our data, it is possible that vmPFC hyperactivity could represent a compensatory response to down-regulate amygdala activity. More broadly, the relationship between greater symptom severity and widespread hyperactivity across fear and emotion circuitry suggests that PTSD symptomatology is associated with greater neural reactivity during extinction learning. This hyperactivity may give rise to aberrant extinction retention.

Our findings suggest that PTSD symptoms and avoidance symptoms in particular are associated with exaggerated fear circuit activity. Previous fear conditioning studies in PTSD have largely focused on reexperiencing and hyperarousal symptom clusters. Reexperiencing symptoms have been demonstrated to be associated with greater fear-potentiated startle during fear acquisition and extinction (Glover et al., 2011; Norrholm et al., 2011). Hyperarousal symptoms, too, are associated with exaggerated fear responding (Jovanovic et al., 2010; Glover et al., 2011; Norrholm et al., 2011). One study reported that in response to script-driven imagery, avoidance symptoms were negatively correlated with vmPFC/rACC activation (Hopper et al., 2007). To our knowledge, however, ours is the first study to demonstrate a link between avoidance symptoms and greater reactivity to cue and context processing. Studies using animal models can provide important insight into the link between avoidance and fear extinction. Some investigators have suggested that the construct of avoidance involves both non-associative novelty fear, which is ameliorated by habituation, and stimulus-specific associative fear, which is ameliorated by extinction training (Pamplona et al., 2011). Generalized avoidance behavior in mice is reduced through both habituation and through extinction training (Costanzi et al., 2011; Pamplona et al., 2011), suggesting that both novelty fear and stimulus-specific fear contribute to avoidance behavior. Our data is consistent with this dual conceptualization, since it demonstrates that avoidance is associated with exaggerated limbic responding to both context (novel) and cues (conditioned). Individuals with greater avoidance symptoms may be more sensitive to both types of stimuli.

The relationship between fear extinction deficits and avoidance symptoms in PTSD might be bidirectional. Previous studies suggest that extinction deficits can lead to the development of avoidance symptoms, and conversely that pre-existent “higher avoidance” can be a contributor to extinction deficits. In support of the first hypothesis, greater fear in response to aversive stimuli is associated with greater levels of subsequent avoidance in rats (Chen et al., 2012). Additionally, pre-trauma deficits in extinction learning are associated with greater risk for developing PTSD after trauma in Dutch soldiers (Lommen et al., 2013). Thus, our findings of greater activity in fear circuits could reflect a mechanism by which individuals develop greater avoidance symptomatology, and provide additional support for the notion of harnessing fear extinction for the purpose of effective avoidance reduction. Alternatively, avoidance could precede fear extinction deficits, in that avoidance symptoms could result in greater fear responding (or amygdala hyperactivity) when confronted with fear-related stimuli that are usually avoided. Theoretical models of PTSD suggest that chronic avoidance leads to greater intensity of avoided cognitions and emotions (Hayes et al., 1999). Additionally, Aderka et al. (2013) recently reported that fear and avoidance predict each other during cognitive-behavioral therapy for social anxiety disorder. Our findings could also reflect resistance to extinction in individuals with greater avoidance. There is evidence to suggest that individuals with greater avoidance have poorer response to CBT (Taylor et al., 2001) and greater rates of attrition (Glynn et al., 1999), though other studies have found that avoidant coping predicts better response to exposure therapy (Leiner et al., 2012). Furthermore, avoidance symptoms may not be as responsive to trauma-focused treatment as other PTSD symptom clusters (Glynn et al., 1999). Longitudinal studies are needed to determine whether fear circuit hyperactivity during extinction is better understood as a risk factor for avoidance symptoms or as a consequence of these symptoms.

Our study had several limitations. First, our paradigm is multimodal in that it involves both visual and tactile cues. However, the conditioned stimuli were primarily visual in nature. Thus, future studies on the relationship between PTSD symptoms and fear conditioning abnormalities could use additional modalities, such as olfactory cues, to further probe the multimodal nature of the link between avoidance symptoms and fear extinction. Second, the design used in this study, i.e., fear conditioning followed by fear extinction, does not allow us to clearly disambiguate the effects of extinction learning from the potential effects of differential recall of CS+ memory trace. For example, the amygdala hyperactivity we observed during extinction may reflect either extinction learning or recall of the CS+ conditioning (see Quirk and Mueller, 2008). Similarly, greater hippocampal activity during both conditioning and extinction learning in avoidant individuals could indicate overconsolidation of fear during conditioning, greater recall of conditioning in the extinction phase, or overgeneralization of fear expression into a neutral context. Future studies could use on-line expectancy ratings to help distinguish between these alternatives. A full factorial design utilizing both new and previously viewed contexts in conjunction with new and previously viewed cues would also be helpful in distinguishing novelty fear from the effects of conditioning. From a clinical perspective, however, PTSD symptoms could be similarly exacerbated by either deficient extinction learning or excessive acquisition-related fear. As such, this phase of extinction may provide a valuable target for research on treatment-enhancing approaches.

In conclusion, our results demonstrate that individuals with greater levels of avoidance exhibit hyperactivation in brain regions involved in fear expression during the presentation of previously conditioned cues and contextual information. This represents a potential brain-based mechanism contributing to the maintenance of fear memories in PTSD patients. Our findings suggest that ameliorating impaired inhibition of fear is an important treatment target for PTSD, in particular for PTSD patients with high levels of avoidance.

## Conflict of Interest Statement

The authors declare that the research was conducted in the absence of any commercial or financial relationships that could be construed as a potential conflict of interest.
